# Catastrophic antiphospholipid syndrome of pregnancy with acute massive cerebral infarction: A case report

**DOI:** 10.1097/MD.0000000000040829

**Published:** 2024-12-13

**Authors:** Xinxing Hu, Meihong Liu

**Affiliations:** a Department of Neurology, Loudi Central Hospital, Loudi, China; b Department of Oncology, Loudi Central Hospital, Loudi, China.

**Keywords:** antiphospholipid antibody syndrome, catastrophic antiphospholipid syndrome, cerebral infarction, pregnancy

## Abstract

**Rationale:**

Catastrophic antiphospholipid syndrome (CAPS) is the most serious type of antiphospholipid antibody syndrome (APS) and can be easily confused with other disorders, such as hemolytic uremic syndrome, disseminated intravascular coagulation and thrombocytopenia syndromes. Timely diagnosis of CAPS poses considerable challenges due to its rarity and the fact that clinicians often lack knowledge of the disease.

**Patient concerns:**

A 21-year-old patient was 32 weeks and 5 days pregnant when she presented to the hospital with a 7-hour history of sudden onset of left-sided limb weakness with no apparent cause. Lupus anticoagulant and/or anticardiolipin antibodies were positive. Head magnetic resonance imaging + magnetic resonance angiography + diffusion weighted imaging: right temporo-occipital insula, right basal ganglia and bilateral radial corona-hemispheric center showed multiple acute-phase cerebral infarction changes and right middle cerebral artery occlusion.

**Diagnoses:**

Catastrophic antiphospholipid syndrome.

**Interventions:**

By intracranial artery thrombectomy and anticoagulation with low-molecular heparin.

**Outcomes:**

The patient’s left limb muscle strength recovered to grade 5. A healthy baby boy was delivered by cesarean section. Both mother and child are safe.

**Lessons:**

The rarity of CAPS is such that misdiagnosis often occurs, culminating in serious complications and even death, emphasizing the need for early recognition, timely diagnosis and immediate treatment. In CAPS that improves with treatment, monitoring and prevention of recurrence is also essential.

## 1. Introduction

Antiphospholipid antibody syndrome (APS) is an autoimmune disorder manifested by frequent vascular thrombotic events, recurrent spontaneous abortions, and thrombocytopenia.^[1]^ Catastrophic Antiphospholipid Syndrome (CAPS) is the most serious type of APS and characterized by rapid onset, poor results, and high mortality.^[1]^ Timely diagnosis of CAPS poses considerable challenges due to its rarity and the fact that clinicians often lack knowledge of the disease. We report a case with catastrophic antiphospholipid syndrome of pregnancy combined with acute massive cerebral infarction to improve the understanding and management of CAPS.

## 2. Case report

On February 11, 2023, a 21-year-old patient, 32 weeks and 5 days pregnant, presented to our hospital with a 7-hour history of sudden left-sided limb weakness without apparent cause. The patient had complete immobility of the left limb and had a history of spontaneous miscarriage. Physical examination: T 36.3°C, BP 133/87 mmHg. The patient’s consciousness and speech were clear, bilateral pupils were about 3 mm in diameter, the light reflex was sensitive, the left nasolabial sulcus became shallow, the angle of the mouth was crooked to the right, and tongue extension was to the left. Her left limb muscle strength was grade 0 with a positive left Babinski sign, and her right limb muscle strength was grade 5 with a negative right Babinski sign. The NIHSS score was 9, and the Modified Rankin Scale score was 5.

**Figure 1.**
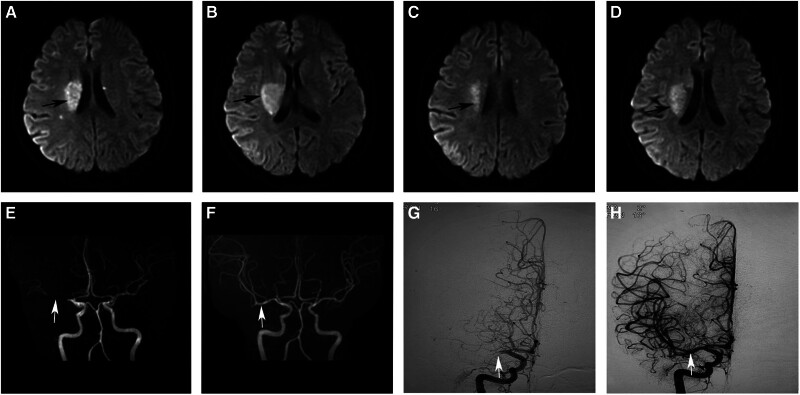
(A and B) Head MRI + DWI: multiple acute-phase cerebral infarctive changes in the right temporo-occipital insula, right basal ganglia, and bilateral radiocoronal-hemispheric centers. (C and D) Head MRI + DWI (reexamination): reduced signal intensity on T2WI and DWI sequences compared to previous. (E) Head MRA: the right middle cerebral artery occlusion. (F) Head MRA (reexamination): the right middle cerebral artery M1 section was visible, and the terminal branches were significantly more visible than before. (G) Cerebral angiogram (preoperative): the right middle cerebral artery M1 was occluded. (H) Cerebral angiography (postoperative): the right middle cerebral artery was patent. DWI = diffusion weighted imaging, MRA = magnetic resonance angiography, MRI = magnetic resonance imaging.

Ancillary tests: white blood cells 12.66 × 10^9^/L, platelets 90 × 10^9^/L, neutrophil ratio 78.2%; D–D polymers > 10 mg/mL; albumin 29.3 g/L, globulin 24.1 g/L; total cholesterol 9.52 mmol/L, triglycerides 3.71 mmol/L, LDL 7.61 mmol/L; urinary routine: occult blood 3+, urinary proteins 2+; lupus antibody primary screening 1 (LA1) 48.30, lupus anticoagulant determination test (LA2) 35.2, LA1/LA2 1.36; anticardiolipin antibody IgM/IgG positive; 24 hours urinary proteins: total protein 3.68 mg/L. Obstetric ultrasound: intrauterine single live fetus (LOA, 31 weeks + 3 days gestation) with normal amniotic fluid and grade 1 placental function. Head magnetic resonance imaging + magnetic resonance angiography + diffusion weighted imaging: right temporo-occipital insula, right basal ganglia, and bilateral radial corona-hemispheric center showed multiple acute phases cerebral infarction changes and right middle cerebral artery occlusion (Fig. 1A, B, and E). ECG: sinus rhythm.

The patient’s condition was serious, to avoid the formation of cerebral edema and other serious complications that threatened the life of the patient, cerebral angiography + intracranial arterial thrombectomy was performed immediately after angiography (Fig. 1G), and 2 pieces of dark-red, tough thrombus (about 3 × 3 × 5 mm in size) were aspirated out by the catheter; re-imaging showed that the right middle cerebral artery was free of blood flow (mTICI grade 3) (Fig. 1H). She was treated postoperatively with low-molecular heparin anticoagulation, infusion of fluids to improve collateral circulation, correction of hypoproteinemia, promotion of fetal lung maturation, and maintenance of water-electrolyte balance. After 3 days, head magnetic resonance imaging + magnetic resonance angiography + diffusion weighted imaging was performed again, and the results showed that the infarct area was reduced compared with the previous 1, the M1 segment of the right middle cerebral artery was well visualized, and the terminal branches were more visible compared with the previous 1 (Fig. 1C, D, and F). The patient’s left limb muscle strength recovered to grade 5. On March 4, 2023, a healthy baby boy was delivered by cesarean section, and the mother and child were safe; postoperative histological examination of the placenta suggested infarction. The diagnosis of CAPS was made based on the patient’s acute onset, positive serology, multi-organ functional impairment (brain, kidney, heart, etc), and histopathologically confirmed thrombosis resulting in cerebral infarction and placental infarction.

## 3. Discussion

CAPS is a rare and severe form of APS occurring in about 1% of APS but with up to 50% mortality. Clinically, CAPS is characterized by multi-organ thrombosis and dysfunction, which may manifest as varying degrees of renal failure, proteinuria, hypertension, pulmonary embolism, stroke, encephalopathy, heart failure, and so on.^[2]^ The various organs showed varying degrees of involvement, with kidney involvement being the most common, accounting for about 73%, followed by lungs (60%), brain (56%), heart (50%), skin (47%), liver (39%), peripheral vasculature (37%), gastro-intestinal (24%), and spleen (18%).^[2]^ CAPS is often difficult to diagnose at an early stage as its clinical manifestations are generally non-characteristic and can be easily confused with other disorders, such as hemolytic uremic syndrome, disseminated intravascular coagulation, and thrombocytopenia syndromes. According to the consensus of international organizations, the following are required for the diagnosis of CAPS: involvement of 3 or more organs, systems, and/or tissues; symptoms occurring simultaneously or within 1 week; histopathological confirmation of occlusion of small blood vessels in at least 1 organ or tissue; and laboratory tests demonstrating the presence of antiphospholipid antibodies (lupus anticoagulant and/or anticardiolipin antibodies).^[3]^ During pregnancy, an additional organ develops in the body, the placenta, so placental thrombosis should also be a consideration in the diagnosis of CAPS.^[4]^

Research data from the International CAPS Registry and the French Multicentre APS/Systemic Lupus Erythematosus Registry suggest that predisposing factors are essential in the development of CAPS,^[2,5]^ with the most common triggers being infections, surgery, malignancy, pregnancy, medications, and lupus flares.^[2]^ In particular, anticoagulants are strongly linked to the development of CAPS. CAPS occurs in more than half of vitamin K-treated APS patients and can be induced by changes in the anticoagulation regimen, studies have shown that the international normalized ratio (INR) in these patients is usually subtherapeutic (<2).^[5]^ Achieving optimal therapeutic levels of INR may be an important part of preventing further progression of APS to CAPS, a finding that should be of great interest to clinical practitioners.

A series of physiological changes during pregnancy or postpartum due to procoagulation, puts the body in a state of hypercoagulability, making it susceptible to venous and even arterial thrombosis, leading to preeclampsia, placenta previa, and recurrent miscarriage.^[6]^ In the past, lack of knowledge about CAPS meant that most pregnant women diagnosed with CAPS already had acute respiratory distress syndrome, alveolar hemorrhage, sepsis, and other critical illnesses, with a maternal mortality rate of around 46% and an infant mortality rate of up to 54%.^[7–9]^ Pregnancy failure rates of 50% or more, despite reductions in mortality rates due to improved screening and early intervention treatments.^[10]^ Hence, CAPS is a high priority for clinicians to be vigilant when pregnant women present with placental dysfunction or other thrombotic events.

As recommended by the guidelines for the management of CAPS, empirical treatment needs to be initiated as soon as CAPS is diagnosed or suspected, with first-line treatment consisting of: elimination of the causative agent (e.g., anti-infection, termination of pregnancy); anticoagulation with low-molecular heparin at a dose of 1 mg/kg every twelve hours; glucocorticosteroids; and plasma exchange.^[4]^ Statistically, the use of the above treatments reduces the mortality rate by 37% and raises the recovery rate by 78%.^[2]^ In addition to the above treatments, intravenous immunogloblin (IVIG) therapy is included in the CAPS Clinical Practice Guideline Programme. There is no consensus on the dose or duration of IVIG administration; it is usually recommended after the last day of plasma exchange, but IVIG should be avoided in the elderly and in those with renal insufficiency, since it leads to an increased risk of adverse renal effects. Two common regimens for IVIG have been proposed: 2 g/kg body weight for 2 to 5 days or 400 mg/kg for 5 days.^[11]^ As each case is individual, the guidelines state that depending on the patient’s condition, first-line treatment may be combined with IVIG or antiplatelet agents as an add-on therapy.^[11]^ Despite significant remissions of clinical manifestations and hematological findings in patients with CAPS after standard treatment, 3.2% of patients were reported to relapse, with a mortality rate of 38%. The researchers found that the most common predisposing factor for relapse was infection, with the brain, kidneys, heart, and lungs being the most commonly affected organs.^[12,13]^ As such, patients with CAPS who have recovered from treatment still need regular checkups to monitor blood markers and avoid infections as a way to improve quality of life and reduce the risk of death.

## 4. Conclusion

CAPS is a clinical emergency that all clinicians need to be aware of, due to its rarity, serious complications and high mortality. Therefore, timely diagnosis and effective treatment of CAPS can greatly reduce the severity of complications and improve the prognosis of patients. At the same time, monitoring and preventing recurrence of CAPS should not be taken lightly.

## Author contributions

**Supervision:** Meihong Liu.

**Writing – original draft:** Xinxing Hu.

**Writing – review & editing:** Xinxing Hu, Meihong Liu.
